# Supplementary material for: Epidemiological and clinical features of hump-nosed pit viper (Hypnale hypnale and Hypnale zara) envenoming in children
Source: PLoS Negl Trop Dis. 2022 Dec 22;16(12):e0011013. doi: 10.1371/journal.pntd.0011013 (PMC9822102; doi:10.1371/journal.pntd.0011013)
Supplement: Attachment — Submitted filename: Answers to the reviewers comments.pdf [file pntd.0011013.s002.pdf]

Answers to the reviewers' comments

"Epidemiological and clinical features of hump-nosed pit viper (*Hypnale hypnale* and *H. zara*) envenoming in children" (PNTD-D-22-01083)

Authors:

R.M.M.K.Namal Rathnayaka,

P.E.Anusha Nishanthi Ranathunga,

S.A.M.Kularatne

2022-10-23

Editor-in-Chief,

PLOS Neglected Tropical Diseases.

Dear Sir,

On behalf of my colleagues, I would like to express my sincere gratitude to you and reviewers for the valuable comments and helpful inputs. Further, we agreed with all the comments raised by the reviewers. Below is our reply and we hope it would satisfy the editors and reviewers. Please find the revised version of our manuscript titled "Epidemiological and clinical features of hump-nosed pit viper (*Hypnale hypnale* and *Hypnale zara*) envenoming in children". All the concerns addressed by the reviewers have been taken into consideration. Please find answers in point by points and all changes are highlighted in red colour in the manuscript. Finally, we hope that the revised version of the manuscript meets the quality standards for publication in your prestigious journal. We are looking forward to hearing from you at your earliest convenience.

Line:
32-33 & 81-83: Should it be ‘latter’ or ‘former’?
Answer:
latter is correct

85-86: Is it ‘biting pattern’?
Answer:
This was corrected as ‘biting frequency’
and was incorporated in to the manuscript (Line 85)

165: Is it ‘mean age’
Answer:
This is median age.
mentioned as median (IQR)
This was incorporated in to the manuscript (Line 170)

264: it should be ‘medial aspect of right foot’
Answer:
This was corrected and was incorporated in to the manuscript (Line 269)

267: ‘ligated about 30 mints’ – not clear
Answer:
This was reworded as,
a tourniquet was applied above the fang punctures for 30 minutes.
This was incorporated in to the manuscript (Line 272)

271: ‘local swelling got severed’ – not clear
Answer:
This was reworded as,
her local swelling increased with prolonging CRFT
and was incorporated in to the manuscript (Line 277)

282: better be ‘bite to dorsum of his right hand’
Answer:
This was corrected (Line 287)

298: spelling ‘haemorrhagic’
Answer:

This was corrected (Line 307)

315-317: needs rephrasing with attention to grammar
Answer:
This was rephrased as follows,

Like in adults, HNPV bites are the commonest cause of snakebites in children which is 44% [7]. In current study, it was 56% of all snakebites.

and was incorporated in to the manuscript (Line 324-326)

317 and 328: has almost similar statement repeated
Answer:
The following statement was omitted from the manuscript
In adults, systemic manifestations following HNPV bites occur in 3.5-39% of patients [2],[3],[4],[5],[13].

338-339: Any cause to explain the longer hospitalization in children?
Answer:
One reason could be the difficulty in clinical assessment in very young children, below the age of 4 years.

This was incorporated as, (Lines 345-347)
One reason for this could be due to the difficulty in clinical assessment of younger children below the age of 4 years.

Method: Was coagulopathy assessed only on admission or followed later on; routinely or during discharge? The consumption coagulopathy may take time to develop and to resolve.
Answer:
The coagulopathy was assessed both on admission and later followed up
The following statements were added to Methods.

All patients were followed for the detection of consumption coagulopathy at 6-hour intervals of WBCT20 for 1-2 days and monitoring of any bleeding manifestation. On admission, clotting profile and WBCT20 were performed. When positive findings were detected clotting profile was done daily for some patients.

This was incorporated in to the manuscript (Lines 123-127)

Case 2: it is not stated what was the PT in this patient before fasciotomy was done.
Answer:
Before the fasciotomy, PT was 13.2/12 sec.

A separate Table was added including important lab findings of these 3 patients who underwent fasciotomy. We hope this may be useful for the readers.
Line 281 **Table 5:** Laboratory findings of patients who underwent fasciotomy

	**Patient1**			**Patient 2**			**Patient 3**		
**Laboratory finding**	**Day 1**	**Day 2**	**Day 3**	**Day 1**	**Day 2**	**Day 3**	**Day 1**	**Day 2**	**Day 3**
WBC ($x10^3$/μL)	9	7	7	16	18	13	11	9	9
Neutrophils (%)	81	52	38	47	42	37	82	59	37
Neutrophil count (/μL)	6870	3750	2600	6910	4110	4190	8810	5300	3270
Platelets ($x10^3$/μL)	301	167	280	287	350	531	411	399	321
Hb (g/dL)	13.1	10.9	12.3	11.7	12.5	12.3	10.8	10.6	12.8
PT (sec)	13/12	14.8/12	15/12	11.8/12	13.2/12	12.4/12	14.5/12	13/12	
INR	1.09	1.28	1.29	0.98	1.1	1.03	1.21	1.08	
APTT (sec)	31/25	37/25	34/25	42.6/32	37.4/32	35/32	28/30	21/30	
Na^+ (mmol/L)	141	140		138	142	141	142	145	136
K^+(mmol/L)	4.3	4.1		4.3	4.1	4.3	4.1	4.7	4.5
Blood urea (mmol/L)	2.3	2.6		3.6	2.5	4	3	1.3	
Creatinine (μmol/L)	37	47		47	34	47	49	38	45
SGOT(AST) [U/I]	34			49			41		
SGPT(ALT) [U/I]	20			16			14		
CRP (mg/L)	< 5			< 5		< 5	< 5	< 6	< 6
WBCT20 (min)	< 20	< 20		< 20			< 20		

Table 1: needs several corrections in 'time of bite' 'transfer from other hospital' section.
Answer:
These were corrected in Table 1

Table 2: Could not understand the difference ‘compartment syndrome leading to fasciotomy and ‘tension releasing incision’

Answer:

There were 2 patients who had small tiny necrosis at the site of bite with moderate swelling. Surgical team decided to make a small subcutaneous incision (about 1cm) on the site of bite for these patients. We called this ‘tension releasing incision’. Now we can understand that both fasciotomy and ‘tension releasing incision’ are same. Therefore, this was removed from the Table in order to avoid the confusion.

Reviewer #1: This is a prospective observational study.

The authors did not mention whether patients were followed for lab evidences of consumption coagulopathy at specific time intervals. As the 'bite to presentation' time was short, only admission profile will miss the developing coagulopathy. This is of more concern for the patients undergoing surgery.

Statistical design is ok.

Answer:

Actually, we followed all patents for lab evidences of VICC at 6h time intervals.

According to the snakebite management guidelines of the hospital, 20 min whole blood clotting test (WBCT20) and PT/INR were performed on admission and then WBCT20 6 hourly

If positive findings are detected, PT/INR are performed frequently according to the severity of the patient. This is mainly due to the resource poor setting.

In Methods, following statements were included.

All patients were followed for the detection of consumption coagulopathy at 6-hour intervals of WBCT20 for 1-2 days and monitoring of any bleeding manifestation. On admission, clotting profile and WBCT20 were performed. When positive findings were detected clotting profile was done daily for some patients.

Reviewer #1: Needs correction of grammatical mistakes.
Few tables need corrections (stated below).
Answer:

These were corrected.
Reviewer #1: Limitations and conclusions are not stated separately.

Answer:

Limitations and conclusions were stated separately in the manuscript as follows,

Line 389-392

Limitations

We encountered difficulty in pain assessment (mild, moderate and severe) as the one of main local envenoming effects in HNPV bites in children below the age of 3 years (n=8).

Line 393-398

Conclusions

Hump-nosed pit viper bites mostly cause local effects and rarely systemic envenoming in children. Compartment syndrome is common in children following their bites. and it is highlighted that these children should be closely monitored in order to detect acute compartment syndrome early and to prevent permanent loss of limbs

Reviewer #2:

The study is relevant and simple. Yes, paediatric envenomations by the Hypnale spp are not well recorded. The quality of English could improve but it is generally clear. The process of identification of patients transferred in with identification of the snake by non-experts is a potential grey area due to misidentification which in turn skews data and its interpretation given that there are at least two species in question in this study. This may be a limitation of the study.

Answer:

Actually, all snakes were identified by the medical officers who have some experience of identifying snakes.

Out of 40, 26 were correctly identified at the species level by the corresponding author. Other 14 (transferred patients from local hospitals) were identified as hump-nosed pit vipers (*Hypnale spp*.) because they were identified by the admitting medical officers of relevant local hospitals (not by the non-experts).

At the same time, data analysis was done with regard to hump-nosed pit viper (Genus: *Hypnale*) bites. (not the separate species)

Thank you,

Yours,

Dr.R.M.M.K.Namal Rathnayaka,
Corresponding Author.

Department of Pharmacology,

Faculty of Medicine,

Sabaragamuwa University of Sri Lanka
