# Supplementary material for: Epidemiological and clinical features of hump-nosed pit viper (Hypnale hypnale and Hypnale zara) envenoming in children
Source: PLoS Negl Trop Dis. 2022 Dec 22;16(12):e0011013. doi: 10.1371/journal.pntd.0011013 (PMC9822102; doi:10.1371/journal.pntd.0011013)
Supplement: Attachment — Submitted filename: Comments PLOS snakebite.docx [file pntd.0011013.s001.docx]

 "Epidemiological and clinical features of hump-nosed pit viper (Hypnale hypnale and H. zara) envenoming in children" (PNTD-D-22-01083)

Line:

32-33 & 81-83: Should it be ‘latter’ or ‘former’?

85-86: Is it ‘biting pattern’?

165: Is it ‘mean age’

264: it should be ‘medial aspect of right foot’

267: ‘ligated about 30 mints’ – not clear

271: ‘local swelling got severed’ – not clear

282: better be ‘bite to dorsum of his right hand’

298: spelling ‘haemorrhagic’

315-317: needs rephrasing with attention to grammar

317 and 328: has almost similar statement repeated

338-339: Any cause to explain the longer hospitalization in children?

Method: Was coagulopathy assessed only on admission or followed later on; routinely or during discharge? The consumption coagulopathy may take time to develop and to resolve.

Case 2: it is not stated what was the PT in this patient before fasciotomy was done.

Table 1: needs several corrections in ‘time of bite’ ‘transfer from other hospital’ section.

Table 2: Could not understand the difference ‘compartment syndrome leading to fasciotomy and ‘tension releasing incision’
